# Extreme heat exposure and depressive symptoms among adolescents: evidence from China

**DOI:** 10.3389/fpsyt.2026.1910951

**Published:** 2026-08-14

**Authors:** Junjie Wang, Qi-fei Xia, Yaocheng Liu, Jiawei Chen

**Affiliations:** 1 School of Physical Education, Ankang College, Ankang, China; 2 School of Physical Education, Southwest University, Chongqing, China; 3 School of Physical Education, Hunan Mechanical Electrical Polytechnic, Changsha, Hunan, China

**Keywords:** adolescents, depressive symptoms, extreme heat exposure, physical exercise, self-rated health, sleep duration

## Abstract

**Background:**

Frequent extreme heat events have become a prominent feature of climate change. However, their potential impact on adolescent mental health and the underlying pathways remain unclear. This study aimed to examine the association between extreme heat exposure and depressive symptoms among Chinese adolescents, and to explore whether sleep duration, physical exercise, and self-rated health may represent potential behavioral and health-related pathways underlying this association.

**Methods:**

This study used adolescent samples from four waves of the China Family Panel Studies (CFPS) conducted between 2016 and 2022. Individual-level CFPS data were matched with the standardized extreme heat exposure index, namely extreme hot days (HTD), from the Chinese Climate Physical Risk Index (CCPRI). Regression models were used to examine the association between HTD and depressive symptoms among adolescents.

**Results:**

Baseline regression results showed that HTD was significantly and positively associated with higher levels of depressive symptoms among adolescents. This association remained robust across a series of sensitivity analyses. Exploratory pathway analyses suggested that HTD was associated with shorter sleep duration, reduced participation in physical exercise, and lower levels of self-rated health, all of which were in turn associated with higher depressive symptom scores. Heterogeneity analyses showed that the association was more pronounced among rural adolescents and male adolescents.

**Conclusion:**

Extreme heat exposure was significantly associated with increased depressive symptoms among Chinese adolescents. Sleep duration, physical exercise, and self-rated health may represent important behavioral and health-related pathways linking extreme heat exposure to adolescent depressive symptoms. These findings suggest that schools, families, and public health agencies should incorporate extreme heat into adolescent mental health risk management and provide targeted support for vulnerable groups, particularly rural and male adolescents.

## 1 Introduction

As early as 2009, the Lancet Commission stated that climate change is “the biggest global health threat of the 21st century” (1). The 2022 Lancet Countdown report further emphasized that climate change continues to threaten population health through multiple pathways, including heat exposure, extreme weather events, and infectious disease risks (2). A growing body of evidence has shown that extreme climate events can substantially impair human well-being and both physical and mental health (3). Among various climate-related hazards, extreme heat is particularly relevant because it directly affects daily sleep, outdoor activity, physical comfort, and emotional regulation. The World Meteorological Organization projected in 2026 that global temperatures from 2026 to 2030 will remain at or near record-high levels, with the frequency and intensity of extreme heat events expected to increase further (4). Rising global temperatures not only affect ecosystems but also harm mental health through pathways such as heat stress, sleep disruption, and restrictions on outdoor activities (5). As a direct environmental stressor, extreme heat has been shown to be significantly associated with increased risks of depression, anxiety, and suicide (6, 7). Adolescents may represent an important vulnerable group in the mental health impacts of climate change (8). These heat-related effects may be especially important for adolescents, whose physiological adaptation, emotion regulation, and self-protection capacities are still developing. A global survey of 10,000 young people aged 16–25 years across 10 countries found that more than 45% of respondents reported that climate-related anxiety had negatively affected their daily lives (9), suggesting that the mental health threat posed by extreme climate events to young people should not be overlooked. However, existing studies have mainly focused on adults in high-income countries, and empirical evidence specifically examining the association between extreme heat exposure and depressive symptoms among Chinese adolescents remains limited. Given China’s large adolescent population and diverse climatic conditions, systematically investigating this issue using nationally representative longitudinal data has important scientific and public health significance.

Depression is one of the most burdensome mental disorders worldwide. The World Health Organization estimated that more than 300 million people worldwide were living with depression in 2015 (10). Projections of the global burden of disease have also suggested that unipolar depressive disorder will remain one of the major contributors to global disease burden by 2030 (11). In China, Lu et al. found, based on a national epidemiological survey, that the proportion of patients with depressive disorders receiving adequate treatment remains low, indicating a substantial treatment gap in depression prevention and care (12). Depressive symptoms do not occur entirely at random and may show certain seasonal patterns. Rosenthal et al. first systematically described seasonal affective disorder, a syndrome characterized by recurrent depressive episodes during specific seasons, and linked it to changes in light exposure and seasonal rhythms (13). This evidence suggests that climatic and environmental conditions may be important external factors associated with depressive symptoms. Against the backdrop of sustained global warming and increasingly frequent extreme heat events, the association between heat exposure as a direct environmental stressor and depressive symptoms warrants systematic investigation.

Previous studies have established links between heat exposure and affective disorders such as depression from multiple perspectives. Using large-scale survey data from the United States, Obradovich et al. found that unusually high temperatures were significantly associated with poorer self-reported mental health (14). Burke et al., using mortality records from the United States and Mexico, found that increases in monthly mean temperature were significantly associated with higher suicide rates, suggesting that heat may intensify mental health risks through pathways such as impaired emotion regulation (15). A systematic review and meta-analysis by Thompson et al. further confirmed that higher ambient temperature was statistically associated with multiple mental health outcomes, including suicide, psychiatric service use, and poorer community mental health (6). In terms of potential pathways, the effects of heat on depressive symptoms may involve several behavioral and health-related processes. Minor et al., using wearable-device data from multiple countries, found that higher nighttime temperatures were significantly associated with shorter sleep duration (16). Li et al., based on more than 23 million repeated sleep records in China, found that climate warming may continuously undermine both sleep duration and sleep quality (17). In addition, hot weather may restrict participation in physical exercise, thereby weakening the protective effect of exercise against depressive symptoms (18, 19). Hou et al., using data from the China Family Panel Studies, found that heat exposure significantly impaired residents’ mental health, with sleep quality and physical health status serving as important intermediate pathways (20). Hua et al. similarly found that rising temperature was significantly associated with poorer mental health among Chinese residents, with stronger effects observed in certain vulnerable groups (21). These studies provide an important foundation for understanding the association between heat exposure and depressive symptoms.

According to the stress-vulnerability model and developmental theories emphasizing adolescence as a sensitive period, adolescents’ self-regulation capacity and emotion regulation strategies are still developing. They may therefore be more psychologically vulnerable when exposed to external environmental stressors (22, 23). As a persistent environmental stressor, extreme heat may have disproportionate adverse effects on the mental health of adolescents who are in a developmentally sensitive stage. The World Health Organization and the United Nations Children’s Fund have reported that approximately one in seven adolescents aged 10–19 years worldwide experiences mental health problems, with depression and anxiety being among the major contributors to adolescent mental health burden (24, 25). In recent years, a small but growing number of studies have begun to examine the association between heat exposure and adolescent mental health. Hao et al., using data from the China Family Panel Studies from 2010 to 2014, found that higher average temperature exposure was significantly associated with poorer mental health among Chinese adolescents, and that adolescents from lower socioeconomic backgrounds were more sensitive to temperature changes (26). Recent longitudinal evidence from Chinese adolescents also suggests that depressive symptoms are dynamically linked with the recall of adverse childhood experiences, highlighting the importance of using longitudinal designs to understand adolescent mental health risks (27, 28). Hu et al., based on cross-sectional data from the Chinese Adolescent Health Survey, found that heatwave exposure was significantly associated with increased risks of depression, anxiety, and their comorbidity among adolescents, with variations across sex and urban-rural subgroups (29). Essers et al., using two European birth cohorts, further found that ambient temperature exposure was associated with psychiatric symptoms, including internalizing problems, externalizing problems, and attention problems among adolescents, although response patterns varied across regions and symptom domains (30).

Sleep, physical exercise, and self-rated health may represent important modifiable pathways linking extreme heat exposure to depressive symptoms among adolescents. Sleep is a fundamental basis for emotional regulation and physical and psychological recovery during adolescence. Systematic reviews and meta-analyses have shown a stable longitudinal association between disturbed sleep and later depressive symptoms in children and adolescents (31). Physical exercise is also an important protective factor for mental health. Systematic reviews and meta-analyses have indicated that physical exercise interventions can alleviate depressive symptoms in children and adolescents, and that higher levels of physical exercise are associated with a lower risk of adolescent depression (32, 33). Self-rated health, as a comprehensive indicator of perceived health status, is also closely related to depressive symptoms among adolescents (34, 35).

**Figure 1:**
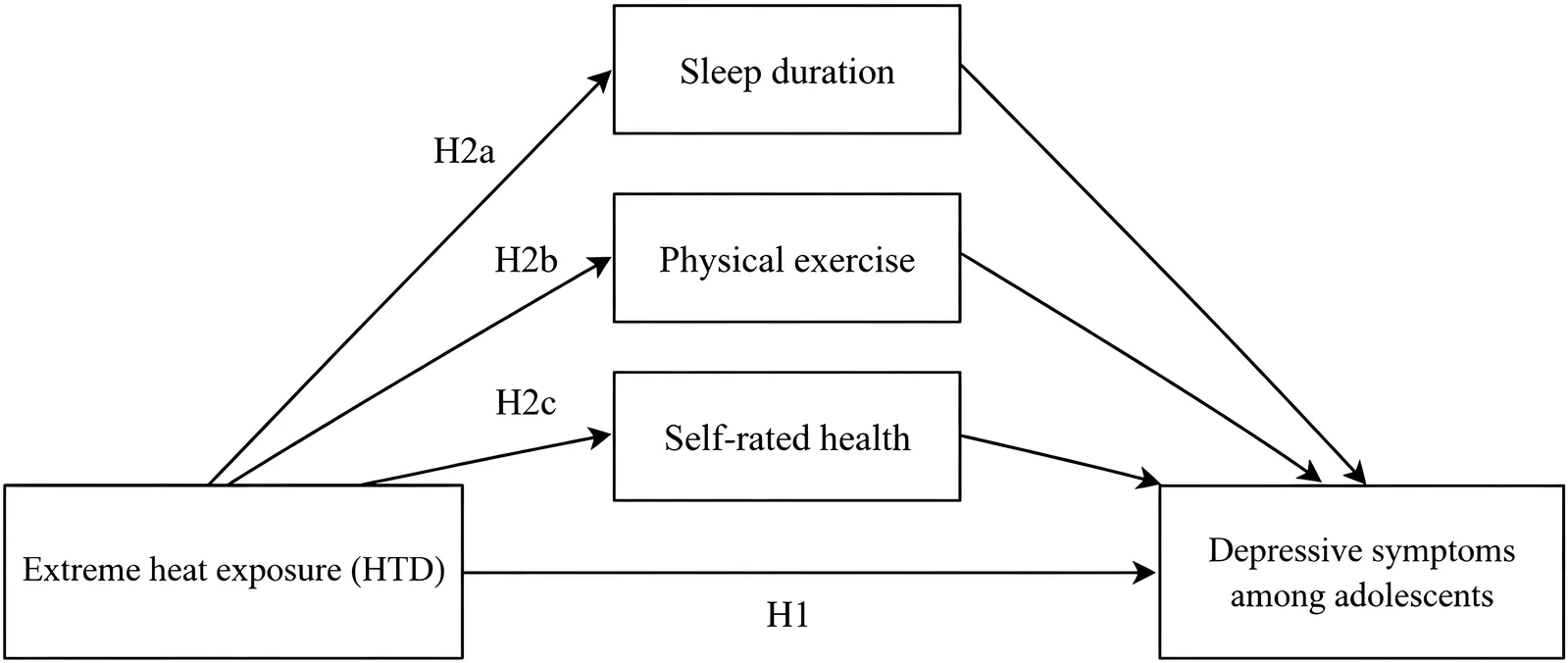
Theoretical framework. H1 indicates the association between extreme heat exposure and depressive symptoms. H2a, H2b, and H2c indicate the proposed potential pathways involving sleep duration, physical exercise, and self-rated health, respectively.

To address these research gaps, this study used CFPS data from 2016, 2018, 2020, and 2022 to construct an unbalanced individual-year sample of adolescents aged 10–19 years. These data were matched with a province-year-level standardized extreme heat exposure index, namely extreme hot days (HTD), to systematically examine the association between extreme heat exposure and depressive symptoms among Chinese adolescents. The objectives of this study were: (1) to examine the association between extreme heat exposure and depressive symptoms among Chinese adolescents; and (2) to test whether sleep duration, physical exercise, and self-rated health constitute potential pathways underlying this association. The hypotheses were as follows: (H1) higher levels of extreme heat exposure are significantly and positively associated with increased depressive symptoms among adolescents; (H2a) sleep duration may represent a potential pathway linking extreme heat exposure to adolescent depressive symptoms; (H2b) physical exercise may represent a potential pathway linking extreme heat exposure to adolescent depressive symptoms; and (H2c) self-rated health may represent a potential pathway linking extreme heat exposure to adolescent depressive symptoms. Based on these hypotheses, this study constructed a conceptual pathway framework involving sleep duration, physical exercise, and self-rated health. It further identified key vulnerable groups by urban-rural residence and sex, and discussed the relevance of self-rated health as a potential health-related vulnerability factor, with the aim of providing empirical evidence for targeted adolescent mental health interventions in the context of climate change. **Figure 1** illustrates the conceptual framework of this study.

## 2 Materials and methods

### 2.1 Data sources and sample selection

**Figure 2:**
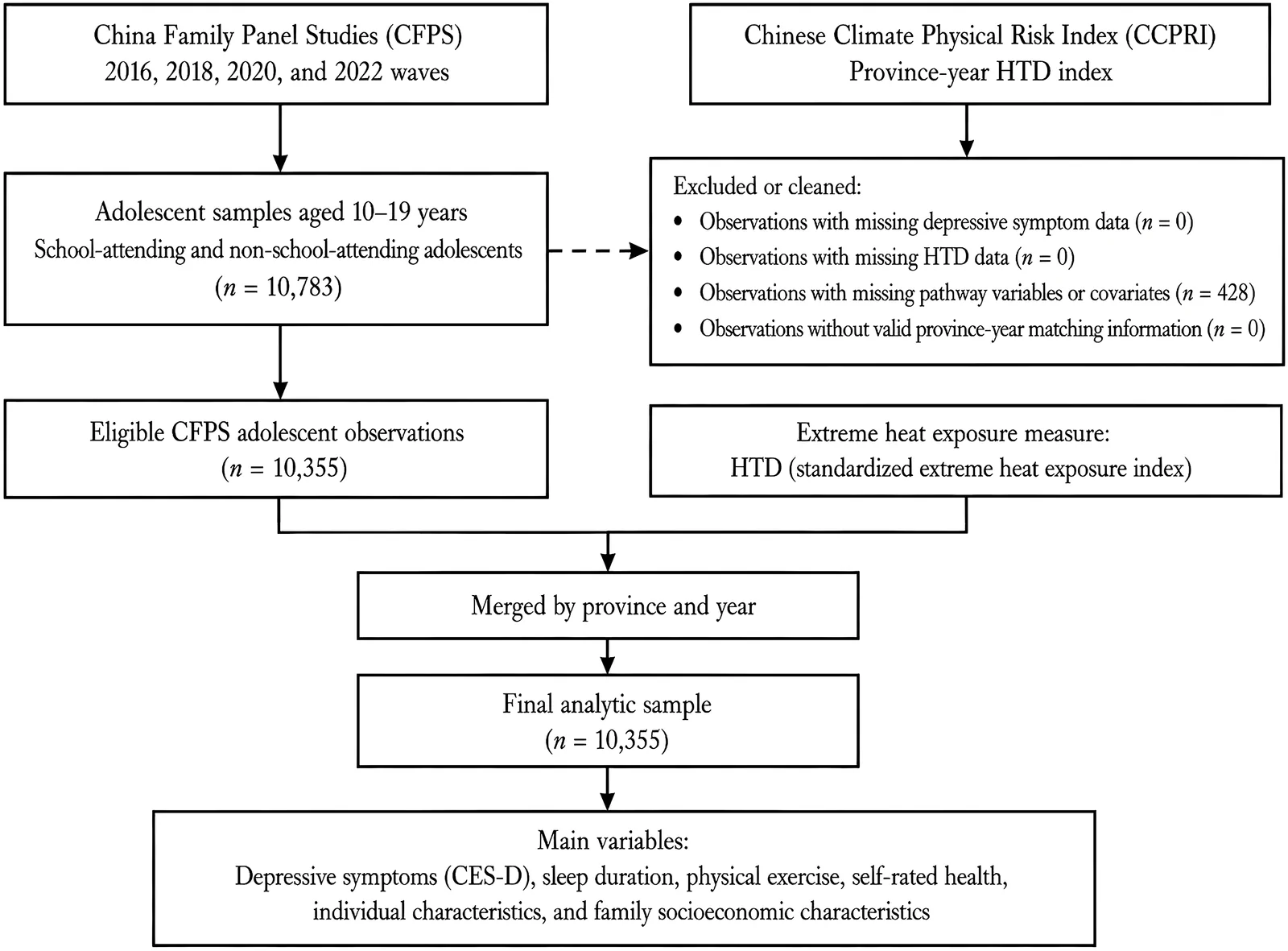
Flowchart of sample inclusion and exclusion.

To examine the association between extreme heat exposure and depressive symptoms among Chinese adolescents, this study used two data sources. Individual-level data were obtained from the China Family Panel Studies (CFPS). The CFPS is a large-scale, nationally representative longitudinal survey conducted by the Institute of Social Science Survey at Peking University. It systematically collects information on households and individuals, including demographics, education, health, and economic conditions (36, 37). This study selected adolescent samples from four survey waves conducted in 2016, 2018, 2020, and 2022. The sample included both school-attending and non-school-attending adolescents aged 10–19 years. Key variables extracted from the CFPS included depressive symptoms, sleep duration, physical exercise, self-rated health, and family socioeconomic characteristics. The data processing and sample selection procedure are illustrated in **Figure 2**.

Climate exposure data were obtained from the Chinese Climate Physical Risk Index (CCPRI). This dataset is constructed based on daily meteorological observations from weather stations and provides extreme climate risk indicators at both the provincial and city levels. These indicators include extreme hot days (HTD), extreme low temperature days (LTD), extreme rainfall days (ERD), and extreme drought days (EDD) (38). In this study, the province-level HTD index corresponding to the CFPS survey years was extracted as the core climate exposure indicator. During data merging, province and year were used as matching keys to link the province-year-level HTD index with the corresponding individual-level adolescent samples. The final analytic sample included 10,355 valid observations.

### 2.2 Measures

#### 2.2.1 Depressive symptoms

The dependent variable in this study was adolescent depressive symptoms, measured using the Center for Epidemiologic Studies Depression Scale (CES-D) in the CFPS data (39). The CES-D scale was developed by Radloff (1977) and is one of the most widely used screening tools for depressive symptoms in epidemiological studies. It has also been widely applied in mental health research among Chinese populations (40, 41).

Since 2016, the CFPS has gradually shifted from the 20-item CES-D to the shortened 8-item CES-D scale (CES-D8). From 2018 onward, all survey waves adopted the CES-D8. The CES-D8 includes eight items covering depressed mood, feeling that everything was an effort, restless sleep, feeling happy, feeling lonely, enjoying life, feeling sad, and feeling that life could not continue. Respondents were asked to report their emotional experiences during the past week. Each item was scored according to frequency: rarely or none of the time (less than 1 day) = 1, some of the time (1–2 days) = 2, often (3–4 days) = 3, and most or all of the time (5–7 days) = 4. The two positive affect items, “feeling happy” and “enjoying life,” were reverse coded. The final score was obtained by summing the eight items.

This study used the variable cesd20sc released in the CFPS data as the measure of depressive symptoms. This variable was converted by the CFPS data team from the CES-D8 score to a score comparable with the 20-item CES-D scale using equipercentile equating, thereby ensuring comparability across the 2016, 2018, 2020, and 2022 survey waves. Because the original CFPS items were scored from 1 to 4 and then converted to a 20-item equivalent score, the actual range of cesd20sc differs from that reported in the standard CES-D literature. Descriptive statistics showed that the mean depressive symptom score among adolescents in the sample was 30.412, with a standard deviation of 6.353, a minimum value of 20, and a maximum value of 70. Higher scores indicate more severe depressive symptoms.

#### 2.2.2 Core explanatory variable

The core explanatory variable in this study was extreme heat exposure, measured using the standardized extreme heat exposure index, namely extreme hot days (HTD), from the Chinese Climate Physical Risk Index (CCPRI). The CCPRI is constructed based on daily meteorological observations from weather stations and systematically quantifies multiple types of climate physical risks across regions, including extreme low temperature days (LTD), extreme hot days (HTD), extreme rainfall days (ERD), and extreme drought days (EDD). These indicators are further combined into the composite Climate Physical Risk Index (CPRI) (36).

It should be noted that the HTD used in this study is not a raw count of hot days, but a standardized relative exposure indicator. The construction of the HTD index follows the following logic: extreme heat events are first identified according to the historical temperature distribution observed at meteorological stations; these events are then aggregated and standardized at the province-year level to generate a relative exposure indicator that is comparable across regions. Therefore, HTD reflects the level of extreme heat exposure in a given province and year relative to its historical baseline. Because the HTD index was measured at the province-year level, it should be interpreted as a regional relative extreme heat risk indicator rather than as a measure of individual-level or location-specific heat exposure. A higher value indicates a stronger relative risk of extreme heat exposure in that province-year.

#### 2.2.3 Potential pathway variables

First, sleep duration was measured using adolescents’ self-reported sleep time. Specifically, this study used responses to the CFPS question “How many hours do you sleep on average each day?” In the exploratory pathway analysis, the original number of sleep hours was log-transformed to reduce the influence of extreme values and improve the residual distribution. After transformation, the mean value was 2.108 and the standard deviation was 0.152.

Second, physical exercise was measured using exercise frequency in the CFPS. The types of physical exercise covered in the CFPS are diverse, including periodic activities such as walking, long-distance running, jogging, and hiking; ball games such as football, basketball, and table tennis; and water-based activities such as swimming, sailing, and diving. The American College of Sports Medicine (ACSM) recommends engaging in moderate physical exercise at least three times per week, with each session lasting 30 minutes or longer. Based on the CFPS question on exercise frequency, this study constructed an ordered categorical variable (exercise_cat), ranging from 0 to 3. The four categories correspond to no exercise, occasional exercise, relatively frequent exercise, and exercise three or more times per week.

Third, health status was measured using self-rated health. Self-rated health reflects individuals’ overall perception of their current health status and is also considered a useful predictor of future health. It has been widely used in previous research. This study used responses to the CFPS question “How would you rate your health status?” The original variable (health_good) ranged from 1 to 5, corresponding to “very unhealthy,” “relatively unhealthy,” “fair,” “relatively healthy,” and “very healthy.” A higher score indicates better self-rated health.

### 2.3 Statistical analysis

#### 2.3.1 Control variables

Individual-level control variables included age, age squared, sex (female = 1, male = 0), years of education, and body mass index (BMI). Family-level control variables included urban-rural residence (urban = 1, rural = 0), the logarithm of annual household income, the logarithm of total household assets, and family size.

Province fixed effects and year fixed effects were included in all regression models. Province fixed effects were used to control for time-invariant geographic, economic, and cultural differences across provinces, such as climate zones, economic development levels, and the allocation of medical resources. Year fixed effects were used to control for macro-level shocks common to all survey years, such as national policy changes and economic cycle fluctuations. Under this two-way fixed effects framework, the identification of HTD comes from within-province changes in extreme heat exposure across different years.

Because the core explanatory variable, HTD, was measured at the province-year level, standard errors were mainly clustered at the province level to account for potential within-province correlation among observations. Standard errors clustered at the individual level were reported as a robustness check.

#### 2.3.2 Baseline regression model

To examine the association between extreme heat exposure and depressive symptoms among Chinese adolescents, this study estimated a baseline regression model controlling for province fixed effects and year fixed effects. The model was specified as follows:


Depression_ipt=α+βHTD_pt+γControls_ipt+μ_p+λ_t+ϵ_ipt

where (Depression_{ipt}) denotes the level of depressive symptoms of adolescent (i) in province (p) and year (t), measured using the CES-D score in the CFPS. (HTD_{pt}) represents the standardized or indexed extreme heat exposure indicator for province (p) in year (t), reflecting the relative level of extreme heat exposure in that province-year rather than the actual number of hot days. (Controls_{ipt}) denotes a set of control variables, including age, age squared divided by 100, sex, urban-rural residence, years of education, BMI, household income, household assets, and family size. (μ_p) represents province fixed effects, which control for time-invariant regional characteristics across provinces. (λ_t) represents year fixed effects, which control for macro-level shocks and time trends common to all survey years. (ε_{ipt}) is the random error term.

#### 2.3.3 Exploratory pathway analysis model

To further explore possible pathways linking extreme heat exposure to adolescent depressive symptoms, this study conducted exploratory pathway analyses from three perspectives: sleep duration, physical exercise, and self-rated health. These analyses were intended to assess whether HTD was associated with these pathway variables and whether these variables were subsequently associated with depressive symptoms, rather than to estimate formal causal mediation effects. First, the following model was estimated to examine whether extreme heat exposure was significantly associated with each pathway variable:


Pathway_ipt=α+βHTD_pt+γControls_ipt+μ_p+λ_t+ϵ_ipt

where (Pathway_{ipt}) represents, respectively, adolescents’ log-transformed sleep duration, physical exercise participation, and self-rated health. Specifically, sleep duration was measured using sleep_hours, which was calculated as the natural logarithm of the original number of sleep hours. Physical exercise was measured using exercise_cat, and self-rated health was measured using health_good, which reflects adolescents’ subjective assessment of their own health status.

In this model, (β) captures the association between extreme heat exposure and the pathway variable. A significant negative association between HTD and sleep duration would suggest that extreme heat may increase depressive risk by impairing sleep recovery. A significant negative association between HTD and physical exercise would suggest that extreme heat may reduce physical exercise and weaken the protective effect of exercise on mental health. A significant negative association between HTD and self-rated health would suggest that extreme heat may affect adolescents’ psychological status by worsening perceived health.

Next, each pathway variable was added to the depressive symptoms model to examine whether it was associated with depressive symptoms after controlling for HTD and other covariates:


Depression_ip=α+βHTD_pt+θPathway_ipt+γControls_ipt+μ_p+λ_t+ϵ_ipt

where (θ) reflects the association between the pathway variable and adolescent depressive symptoms. If the estimated coefficient of HTD decreases after the pathway variable is added, and the pathway variable is significantly associated with depressive symptoms, this would suggest that the variable may be related to a possible behavioral or health-related pathway linking extreme heat exposure to adolescent depressive symptoms. It should be noted that these analyses do not estimate indirect effects, total effects, or confidence intervals for mediation. Therefore, the findings should be interpreted as exploratory pathway evidence rather than evidence of formal causal mediation. Descriptive statistics for the main variables are presented in **Table 1**.

**Table 1:** Descriptive statistical analysis of variables.

Variable	N	Mean	SD	Min	p50	Max
Cesd20sc	10,355	30.412	6.353	20.000	30.000	70.000
HTD	10,355	71.487	11.791	43.743	70.845	100.519
Sleep	10,355	8.324	1.195	2.000	8.000	16.000
Exercise	10,355	1.793	1.117	0.000	2.000	3.000
Health	10,355	3.978	0.902	1.000	4.000	5.000
Age	10,355	14.637	2.894	10.000	15.000	19.000
Female	10,355	0.470	0.499	0.000	0.000	1.000
Education	10,355	8.085	2.928	0.000	8.000	16.000
BMI	10,355	19.230	3.554	10.000	18.789	54.687
Urban	10,355	0.441	0.497	0.000	0.000	1.000
Family_income	10,355	11.053	0.931	0.000	11.108	13.288
Total_asset	10,355	11.580	3.704	0.000	12.539	15.884
Familysize	10,355	4.966	1.827	1.000	5.000	16.000

## 3 Empirical analysis

### 3.1 Baseline regression results

To examine the association between extreme heat exposure and depressive symptoms among Chinese adolescents, this study estimated the baseline regression model while controlling for province fixed effects and year fixed effects. **Table 2** presents the baseline regression results. The estimated coefficients of the HTD index were positive across all columns and were statistically significant. After all control variables were included, the estimated coefficient of HTD was 0.022 and was significant at the 1% level. This indicates that higher relative exposure to extreme heat was significantly associated with higher levels of depressive symptoms among Chinese adolescents. In terms of effect size, given that the standard deviation of HTD was 11.791 and the standard deviation of depressive symptom scores was 6.353, a one-standard-deviation increase in HTD corresponded to an average increase of approximately 0.26 points in depressive symptom scores, equivalent to about 4.1% of the standard deviation of depressive symptoms. Therefore, although the coefficient of HTD was statistically significant, the magnitude of the association was relatively modest and should be interpreted cautiously. Given that HTD was measured at the province-year level, this result should be interpreted as an association between regional relative extreme heat risk and adolescent depressive symptoms, rather than as evidence of the effect of individual-level heat exposure.

**Table 2:** Baseline regression results.

Variables	(1)	(2)
	Depressive symptoms	Depressive symptoms
HTD index	0.022***	0.022***
	(3.41)	(3.48)
Age		0.769***
		(3.00)
Age squared/100		-0.241
		(-0.29)
Female		0.296**
		(2.15)
Years of education		-0.456***
		(-7.41)
BMI		0.012
		(0.56)
Urban		-0.017
		(-0.12)
Log household income		-0.156**
		(-2.07)
Log household assets		-0.042**
		(-2.42)
Family size		0.079**
		(2.03)
Observations	10,355	10,355
Adjusted R²	0.024	0.046
Province fixed effects	Yes	Yes
Year fixed effects	Yes	Yes

t values are shown in parentheses; ***, **, and * indicate significance at the 1%, 5%, and 10% levels, respectively.

In terms of substantive interpretation, HTD is a standardized or indexed relative exposure indicator. Therefore, its regression coefficient should be interpreted as the statistical association between a one-unit increase in the HTD index and changes in adolescent depressive symptom scores, rather than the effect of an additional actual hot day. Although the marginal association corresponding to a one-unit increase in HTD is relatively small, extreme heat exposure often has persistent, cumulative, and seasonal characteristics, and its association with adolescent mental health risks remains worthy of attention. Adolescents are in a critical stage of physical and psychological development and habit formation. Extreme heat may be associated with their psychological status by increasing physical discomfort, disrupting sleep rhythms, reducing outdoor activities and physical exercise, and lowering perceived health. Thus, Hypothesis H1 was supported.

### 3.2 Robustness checks

#### 3.2.1 Winsorization

To avoid the potential influence of extreme values on the estimation results, this study re-estimated the baseline model after applying 5% winsorization to the main continuous variables. The results are reported in **Table 3**. The regression results showed that, after winsorization, the estimated coefficient of extreme heat exposure, measured by HTD, remained positive and significant at the 1% level. After further including control variables, the estimated coefficient of HTD was 0.016 and remained significant at the 1% level. These results indicate that the positive association between extreme heat exposure and adolescent depressive symptoms persisted even after adjusting for extreme values, suggesting that the baseline findings were not driven by a small number of outliers or extreme observations.

**Table 3:** Robustness check: 5% winsorization.

Variables	(1)	(2)
	Depressive symptoms	Depressive symptoms
HTD index	0.017***	0.016***
	(2.65)	(2.61)
Controls	No	Yes
Observations	10,355	10,355
Adjusted R^2^	0.025	0.047
Province fixed effects	Yes	Yes
Year fixed effects	Yes	Yes

t values are shown in parentheses; ***, **, and * indicate significance at the 1%, 5%, and 10% levels, respectively.

#### 3.2.2 Excluding special city samples

Considering that first-tier cities may influence the estimation results because of their higher levels of economic development, public service provision, and air-conditioning penetration, this study further re-estimated the regression model after excluding samples from first-tier cities. The results are reported in **Table 4**. The results showed that the estimated coefficient of HTD remained positive after these samples were excluded. After control variables were included, the estimated coefficient of HTD was 0.017 and was significant at the 5% level. This finding indicates that the positive association between extreme heat exposure and adolescent depressive symptoms was not driven solely by special city samples such as Beijing, Shanghai, Guangzhou, and Shenzhen. Even after excluding these cities with relatively distinctive economic conditions and public service resources, the positive association between extreme heat exposure and depressive symptoms remained.

**Table 4:** Robustness check: excluding first-tier city samples.

Variables	(1)	(2)
	Depressive symptoms	Depressive symptoms
HTD index	0.016**	0.017**
	(2.22)	(2.37)
Controls	No	Yes
Observations	9,055	9,055
Adjusted R^2^	0.025	0.048
Province fixed effects	Yes	Yes
Year fixed effects	Yes	Yes

t values are shown in parentheses; ***, **, and * indicate significance at the 1%, 5%, and 10% levels, respectively.

#### 3.2.3 Alternative fixed effects specification

To control for unobserved time-invariant factors at the individual level, this study further estimated a model with individual fixed effects and year fixed effects. The results showed that, after controlling for individual fixed effects and year fixed effects, the estimated coefficient of HTD remained positive, at approximately 0.013, and was significant at the 10% level. Compared with the baseline model, the statistical significance was reduced, but the direction of the coefficient remained consistent. This suggests that the positive association between extreme heat exposure and adolescent depressive symptoms remained to some extent robust after more strictly controlling for time-invariant individual characteristics. The results are reported in **Table 5**.

**Table 5:** Robustness check: alternative fixed effects specification.

Variables	(1)	(2)
	Depressive symptoms	Depressive symptoms
HTD index	0.013*	0.013*
	(1.70)	(1.69)
Controls	No	Yes
Observations	10,355	10,355
Adjusted R^2^	0.310	0.309
Province fixed effects	Yes	Yes
Year fixed effects	Yes	Yes

t values are shown in parentheses; ***, **, and * indicate significance at the 1%, 5%, and 10% levels, respectively.

#### 3.2.4 Alternative clustering levels for standard errors

Considering that the core explanatory variable, the HTD index, was obtained by matching climate exposure data at the province-year level, adolescents within the same province may be exposed to similar climate shocks and region-specific disturbances. Therefore, while keeping the sample, control variables, and fixed effects specifications unchanged, this study further clustered standard errors at the province level and compared the results with those obtained using standard errors clustered at the individual level. **Table 6** reports the results based on alternative clustering levels. The estimated coefficients of the HTD index remained significantly positive across specifications. This indicates that the finding of a significant positive association between extreme heat exposure and increased depressive symptoms among adolescents did not depend on a single clustering specification for standard errors.

**Table 6:** Robustness check: alternative clustering levels for standard errors.

Variables	Individual clustering Baseline model	Province clustering Baseline model	Individual clustering Fully controlled model	Province clustering Fully controlled model
HTD index	0.022*** (3.41)	0.022*** (3.11)	0.022*** (3.48)	0.022*** (3.30)
Controls	No	No	Yes	Yes
Province fixed effects	Yes	Yes	Yes	Yes
Year fixed effects	Yes	Yes	Yes	Yes
Observations	10355	10355	10355	10355
Adjusted R^2^	0.024	0.024	0.046	0.046

*** indicates significance at the 1% level.

#### 3.2.5 Robustness check using the CES-D 0–60 scale

To improve the international readability of the depressive symptom measure and to examine whether the results depended on the original scoring method of the scale, this study further converted the original CES-D score in the CFPS to the 0–60 scale that is more commonly used in international studies. Specifically, this study constructed the variable (CESD0_60 = cesd20sc - 20), thereby converting the original 20–80 scoring range to a 0–60 scoring range.

**Table 7** reports the robustness check results using the CES-D 0–60 score as the dependent variable. In the model that controlled only for province fixed effects and year fixed effects, the estimated coefficient of the HTD index was 0.022 and was significant at the 1% level. After including individual characteristics, family socioeconomic characteristics, and all control variables, the estimated coefficient of the HTD index remained positive and statistically significant. In the fully controlled model, for example, the estimated coefficient of HTD was 0.022, with a t value of 3.30 when standard errors were clustered at the province level. These results indicate that the positive association between extreme heat exposure and increased depressive symptoms among adolescents remained stable after converting the depressive symptom score to the 0–60 scale.

**Table 7:** Robustness check: CES-D 0–60 scale conversion.

Variables	(1)	(2)	(3)	(4)
	Baseline model	Individual characteristics model	Family characteristics model	Fully controlled model
HTD index	0.022*** (3.11)	0.023*** (3.47)	0.021*** (2.98)	0.022*** (3.30)
Controls	No	Yes	No	Yes
Observations	10,355	10,355	10,355	10,355
Adjusted R^2^	0.024	0.045	0.025	0.046
Province fixed effects	Yes	Yes	Yes	Yes
Year fixed effects	Yes	Yes	Yes	Yes
Standard error clustering level	Province	Province	Province	Province

t values are shown in parentheses; ***, **, and * indicate significance at the 1%, 5%, and 10% levels, respectively. All models control for province and year fixed effects, with standard errors clustered at the province level.

#### 3.2.6 Robustness check using propensity score matching

**Figure 3:**
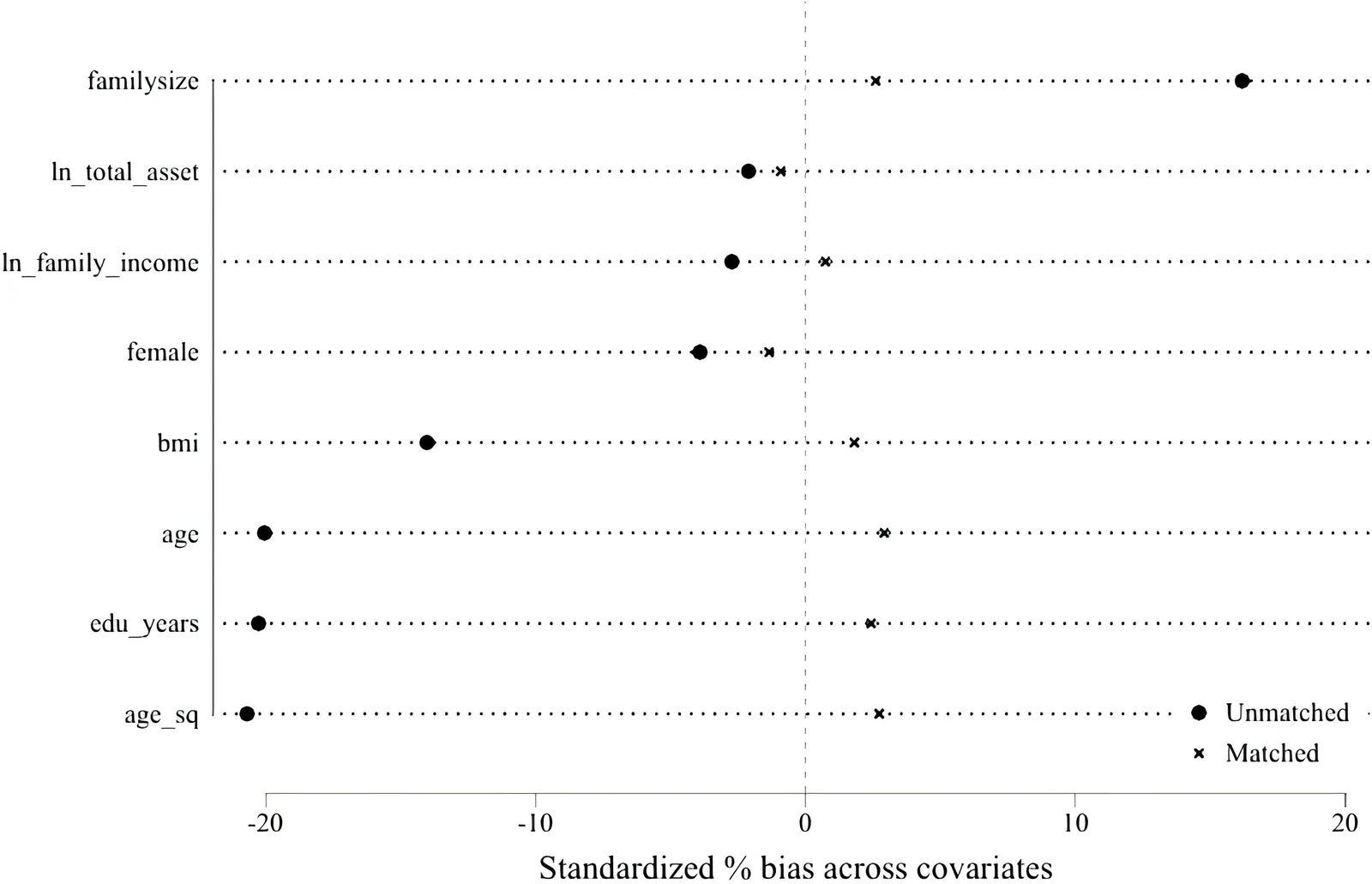
Standardized covariate bias before and after PSM matching.

Finally, considering that adolescents from different regions may differ systematically in individual characteristics, family backgrounds, and living environments, this study further employed propensity score matching (PSM) as an auxiliary robustness check. It should be noted that the HTD index is a continuous climate exposure indicator, whereas PSM typically requires the construction of a binary treatment variable. Therefore, only in this robustness check, the HTD index was dichotomized. Specifically, the sample median of the HTD index, 70.84501, was used as the cutoff. Observations with an HTD index above the median were defined as the high extreme heat exposure group ((psm = 1)), whereas observations with an HTD index less than or equal to the median were defined as the low extreme heat exposure group ((psm = 0)) (**Figure 3**).

**Figure 4:**
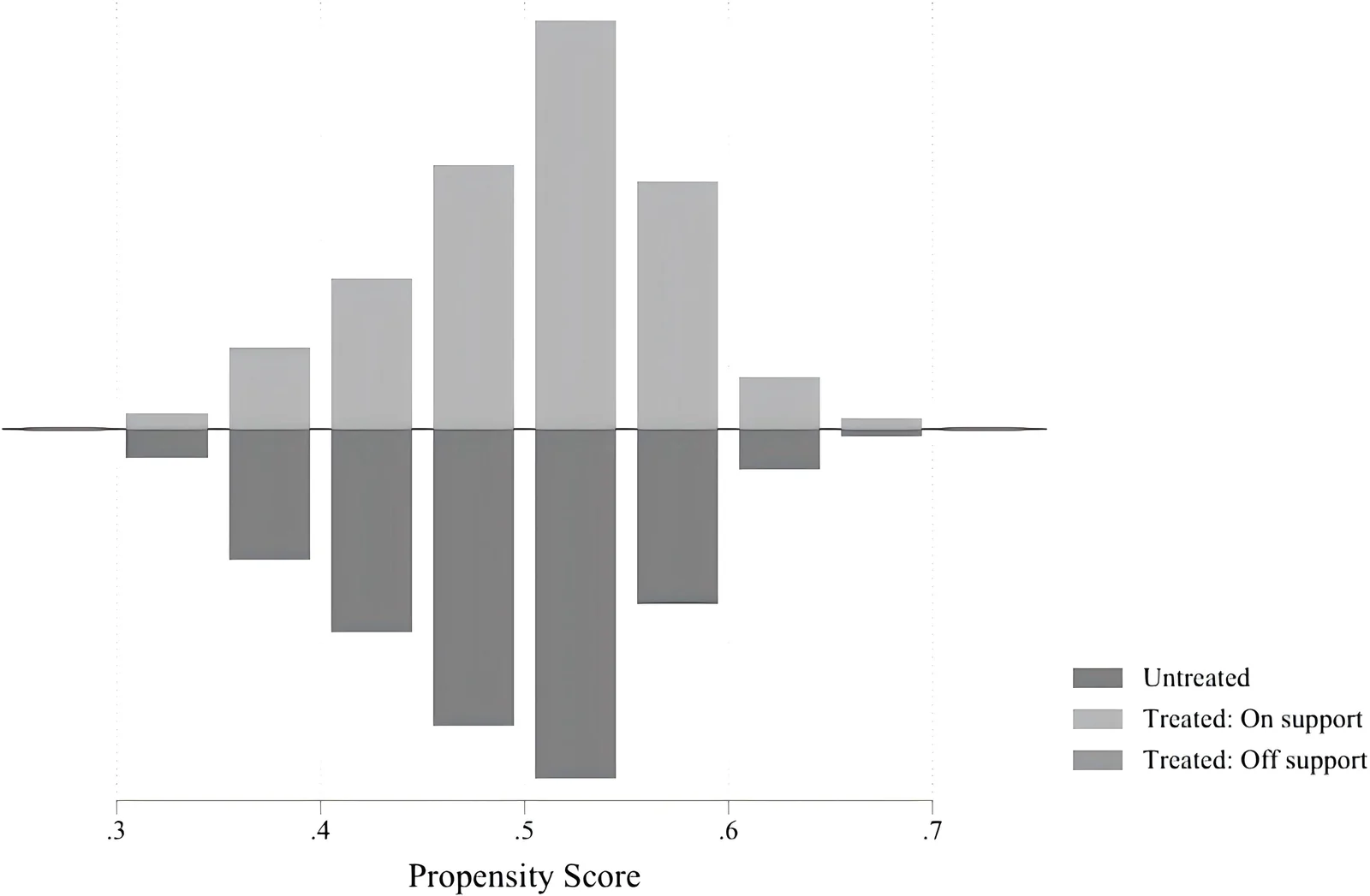
Common support region of propensity scores after PSM matching.

This classification was used only to examine whether the baseline results remained stable after balancing observable characteristics between the high- and low-HTD exposure groups. It did not change the main analytical strategy of this study, in which the continuous HTD index was used as the core explanatory variable. In estimating the propensity scores, this study used nearest-neighbor matching with a caliper of 0.05. The control variables were used as matching covariates, and a Logit model was employed to estimate the probability of entering the high extreme heat exposure group. After matching, observations with missing matching weights were excluded, and the baseline regression model was re-estimated using the matched sample (**Figure 4**).

The regression results based on the matched sample are reported in **Table 8**. After control variables were included, the estimated coefficient of HTD was 0.015 and was significant at the 5% level. These results indicate that, after adjusting for observable differences between the high- and low-extreme-heat exposure groups through matching, the positive association between the HTD index and increased depressive symptoms among adolescents remained.

**Table 8:** Robustness check: propensity score matching (PSM).

Variables	(1)	(2)
	Depressive symptoms	Depressive symptoms
HTD index	0.017**	0.015**
	(2.41)	(2.13)
Controls	No	Yes
Observations	8,159	8,159
Adjusted R^2^	0.025	0.047
Province fixed effects	Yes	Yes
Year fixed effects	Yes	Yes

t values are shown in parentheses; ***, **, and * indicate significance at the 1%, 5%, and 10% levels, respectively.

### 3.3 Exploratory pathway analyses

#### 3.3.1 Sleep duration as a potential pathway

Sleep is an important basis for emotional regulation and psychological recovery among adolescents. Extreme heat, especially nighttime heat, may reduce thermal comfort in the sleep environment, increase difficulty falling asleep and nighttime awakenings, and thereby shorten sleep duration.

As shown in **Table 9**, the exploratory pathway analysis showed that the estimated coefficient of the HTD index for log-transformed sleep duration (sleep_hours) was -0.002 and was significant at the 5% level. This indicates that higher relative exposure to extreme heat was significantly associated with shorter sleep duration among adolescents. Because sleep_hours was expressed in logarithmic form, this result should be interpreted as an association between an increase in the HTD index and a relative decrease in sleep duration, rather than a direct reduction of 0.002 hours in the original number of sleep hours. Furthermore, when log-transformed sleep duration and the HTD index were included simultaneously in the regression model, the estimated coefficient of log-transformed sleep duration for depressive symptoms was -4.091 and was highly significant at the 1% level. This suggests that longer sleep duration was significantly associated with lower levels of depressive symptoms among adolescents. Meanwhile, after controlling for sleep duration, the estimated coefficient of the HTD index for depressive symptoms was 0.020 and remained significant at the 1% level.

**Table 9:** Exploratory pathway analysis: sleep duration.

	(1)	(2)
Variables	Log sleep duration	Depressive symptoms
HTD index	-0.002**	0.020***
	(-2.02)	(3.07)
Log sleep duration		-4.091***
		(-6.18)
Controls	Yes	Yes
Observations	10,355	10,355
Adjusted R^2^	0.128	0.054
Province fixed effects	Yes	Yes
Year fixed effects	Yes	Yes

t values are shown in parentheses; ***, **, and * indicate significance at the 1%, 5%, and 10% levels, respectively.

Taken together, these results suggest that sleep duration may represent a potential pathway linking extreme heat exposure to adolescent depressive symptoms. Specifically, HTD was associated with shorter sleep duration, and shorter sleep duration was associated with higher depressive symptom scores. However, because this study is based on observational data and does not formally estimate indirect effects, these findings should be interpreted as exploratory pathway evidence rather than evidence of a strict causal mediation effect. Overall, the results are consistent with Hypothesis H2a, which proposes sleep duration as a potential pathway linking extreme heat exposure to adolescent depressive symptoms.

#### 3.3.2 Physical exercise as a potential pathway

Physical exercise is an important protective factor for adolescent mental health. Moderate physical exercise may reduce the risk of depressive symptoms by relieving stress, improving mood, enhancing self-efficacy, and promoting social interaction. However, extreme heat increases the physical burden and health risks associated with outdoor activities, which may reduce adolescents’ willingness and opportunities to participate in physical exercise. This may be particularly relevant for running, ball games, outdoor activities, and extracurricular school sports. **Table 10** reports the exploratory pathway analysis for physical exercise.

**Table 10:** Exploratory pathway analysis: physical exercise.

	(1)	(2)
Variables	Physical exercise	Depressive symptoms
HTD index	-0.002**	0.022***
	(-2.03)	(3.25)
Physical exercise		-0.101**
		(-2.01)
Controls	Yes	Yes
Observations	10,355	10,355
Adjusted R^2^	0.139	0.046
Province fixed effects	Yes	Yes
Year fixed effects	Yes	Yes

t values are shown in parentheses; ***, **, and * indicate significance at the 1%, 5%, and 10% levels, respectively.

Furthermore, physical exercise and the HTD index were included simultaneously in the regression model for depressive symptoms. The results showed that the estimated coefficient of physical exercise for depressive symptoms was -0.101 and was significant at the 5% level. This indicates that maintaining or increasing participation in physical exercise was significantly associated with lower levels of depressive symptoms among adolescents, suggesting a positive mental health protective role. Meanwhile, after controlling for physical exercise, the estimated coefficient of the HTD index for depressive symptoms was 0.022 and remained highly significant at the 1% level.

Taken together, these results suggest that physical exercise may represent a potential behavioral pathway linking extreme heat exposure to adolescent depressive symptoms. Specifically, HTD was associated with reduced participation in physical exercise, and lower participation in physical exercise was associated with higher depressive symptom scores. However, given the observational nature of the data and the absence of formal indirect-effect estimation, these results should be interpreted as exploratory pathway evidence rather than evidence of strict causal mediation. Overall, the findings are consistent with the proposed pathway involving physical exercise.

#### 3.3.3 Self-rated health as a potential pathway

Although self-rated health is not an objective physical examination indicator, it reflects individuals’ overall perception of their own health status and represents an important variable linking physical discomfort with psychological status. As shown in **Table 11**, the exploratory pathway analysis showed that the estimated coefficient of the HTD index for self-rated health (health_good) was -0.002 and was significant at the 1% level. Because a higher value of health_good indicates better self-rated health, this result suggests that higher relative exposure to extreme heat was significantly associated with lower levels of self-rated health among adolescents.

**Table 11:** Exploratory pathway analysis: self-rated health.

Variables	(1)	(2)
	Self-rated health	Depressive symptoms
HTD index	-0.002***	0.019***
	(-2.60)	(2.90)
Self-rated health		-1.448***
		(-27.66)
Controls	Yes	Yes
Observations	10,355	10,355
Adjusted R^2^	0.053	0.086
Province fixed effects	Yes	Yes
Year fixed effects	Yes	Yes

t values are shown in parentheses; ***, **, and * indicate significance at the 1%, 5%, and 10% levels, respectively.

Furthermore, self-rated health and the HTD index were included simultaneously in the regression model for depressive symptoms. The results showed that the estimated coefficient of self-rated health for depressive symptoms was -1.448 and was highly significant at the 1% level. This indicates that better self-rated health was strongly associated with lower levels of depressive symptoms among adolescents, suggesting an important protective role for perceived health. Meanwhile, after controlling for self-rated health, the estimated coefficient of the HTD index for depressive symptoms was 0.019 and remained significant at the 1% level.

Taken together, these results suggest that self-rated health may represent another potential health-related pathway linking extreme heat exposure to adolescent depressive symptoms. Specifically, HTD was associated with lower self-rated health, and lower self-rated health was associated with higher depressive symptom scores. However, because self-rated health and depressive symptoms may influence each other, and because this study is based on observational data without formal indirect-effect estimation, these findings should be interpreted as exploratory pathway evidence rather than evidence of strict causal mediation. Overall, the results are consistent with the proposed pathway involving self-rated health.

After log-transformed sleep duration was included in the depressive symptoms model, its estimated coefficient was -4.091 and was significant at the 1% level, indicating that adolescents with longer sleep duration had relatively lower depressive symptom scores. Meanwhile, the estimated coefficient of the HTD index remained positive and statistically significant. After physical exercise was included in the model, its estimated coefficient was -0.101 and was significant at the 5% level, suggesting that higher participation in physical exercise was associated with lower levels of depressive symptoms. After self-rated health was included in the model, its estimated coefficient was -1.448 and was significant at the 1% level, indicating that better self-rated health was associated with lower depressive symptom scores among adolescents. Overall, these results further suggest that sleep duration, physical exercise, and self-rated health were all statistically associated with adolescent depressive symptoms in the expected directions, providing additional exploratory evidence for the proposed pathways corresponding to H2a, H2b, and H2c.

### 3.4 Heterogeneity analysis

#### 3.4.1 Urban-rural heterogeneity

**Table 12** reports the heterogeneity analysis by urban-rural residence. This study divided the sample into urban adolescents and rural adolescents according to urban-rural residence, and separately examined the association between extreme heat exposure and depressive symptoms in the two subsamples. The regression results showed that, in the urban sample, the estimated coefficient of HTD was 0.022 and was significant at the 5% level. In the rural sample, the estimated coefficient of HTD was 0.024 and was also significant at the 5% level. The Chow test indicated a statistically significant difference between the urban and rural groups.

**Table 12:** Heterogeneity analysis: urban-rural differences.

	Urban	Rural
Variables	Depressive symptoms	Depressive symptoms
HTD index	0.022**	0.024**
	(2.51)	(2.53)
Controls	Yes	Yes
Chow test	P=0.00001	P=0.00001
Observations	5,791	4,564
Adjusted R^2^	0.051	0.047
Province fixed effects	Yes	Yes
Year fixed effects	Yes	Yes

t values are shown in parentheses; ***, **, and * indicate significance at the 1%, 5%, and 10% levels, respectively.

These results suggest that extreme heat exposure was significantly and positively associated with depressive symptoms among both urban and rural adolescents, but the estimated coefficient was slightly higher in the rural sample. This may be related to the relatively limited availability of heat adaptation resources in rural areas. Compared with urban areas, some rural households may have weaker access to air conditioning, shaded spaces, medical services, mental health support, and indoor exercise facilities. As a result, rural adolescents may experience more pronounced physical discomfort, sleep disruption, and restrictions on outdoor activities during hot weather. Therefore, rural adolescents may represent a key group requiring attention in relation to extreme heat-related mental health risks.

#### 3.4.2 Sex heterogeneity

**Table 13** reports the heterogeneity analysis by sex. This study further divided the sample into female and male adolescents according to sex. The regression results showed that, in the female sample, the estimated coefficient of HTD was 0.015, but it did not reach conventional levels of statistical significance. In the male sample, the estimated coefficient of HTD was 0.028 and was significant at the 1% level. This indicates that, under the sample and model specifications used in this study, the association between extreme heat exposure and increased depressive symptoms was more pronounced among male adolescents.

**Table 13:** Heterogeneity analysis: sex differences.

Variables	Female	Male
	Depressive symptoms	Depressive symptoms
HTD index	0.015	0.028***
	(1.59)	(3.28)
Controls	Yes	Yes
Observations	4,869	5,486
Adjusted R²	0.053	0.045
Province fixed effects	Yes	Yes
Year fixed effects	Yes	Yes

t values are shown in parentheses; ***, **, and * indicate significance at the 1%, 5%, and 10% levels, respectively.

It should be noted that the positive but statistically non-significant coefficient of HTD in the female sample does not imply that female adolescents were unaffected by heat exposure. Rather, it suggests that, within the present sample and model specification, the estimated association was stronger and more statistically evident among male adolescents.

## 4 Discussion

Based on adolescent samples from the CFPS 2016–2022 waves and the Chinese Climate Physical Risk Index, this study examined the association between extreme heat exposure and depressive symptoms among Chinese adolescents. The results showed that the province-year-level HTD index was significantly associated with higher levels of depressive symptoms among adolescents. This association remained stable after controlling for age, sex, urban-rural residence, years of education, BMI, household income, household assets, family size, province fixed effects, and year fixed effects. A series of robustness checks also yielded consistent results. As reported in the baseline results, the effect size of HTD was statistically significant but modest, with a one-standard-deviation increase in HTD corresponding to approximately 4.1% of the standard deviation of depressive symptom scores.

This finding is broadly consistent with recent studies on heat exposure and adolescent mental health. Existing evidence suggests that climate change and rising ambient temperatures may affect mental health through physical discomfort, sleep disruption, activity restrictions, and psychosocial stress (42). Children and adolescents may be particularly sensitive. Their physical development, emotion regulation, risk perception, and self-protection abilities are still maturing, and their daily lives remain highly dependent on families, schools, and community environments (8, 23). A systematic review and meta-analysis by Lai et al. showed that heat exposure was associated with increased risks of mental health-related hospital visits, depression-related outcomes, and composite mental health problems among children and adolescents (7). Hu et al., using data from the Chinese Adolescent Health Survey, further found that heatwave exposure was associated with increased risks of depression, anxiety, and their comorbidity among adolescents, with males and rural students potentially more vulnerable (29). The findings of the present study are consistent with these studies and provide additional evidence. Compared with previous studies, this study contributes to the literature in several ways. Unlike studies using annual mean temperature or heatwave days, this study used a standardized extreme heat exposure index, which may more precisely capture the intensity and relative level of extreme climate shocks. In addition, the use of four waves of longitudinal data and a two-way fixed effects design allows better control for time-invariant differences at the individual and provincial levels than cross-sectional designs.

The association between extreme heat and depressive symptoms among adolescents may first arise from the combined effects of heat stress on physical and emotional states. High temperatures increase the burden of heat dissipation and may lead to fatigue, irritability, headache, impaired attention, and physical discomfort. Adolescents are at a stage where academic pressure, peer relationships, and physical development intersect, and external heat stress may amplify existing stressors. Heat is not merely a meteorological background factor. It enters daily life: poorer sleep at night, low energy during the day, fewer outdoor activities, reduced learning efficiency, physical discomfort, and greater emotional fluctuation. These changes may not immediately manifest as clinical disorders, but they may be reflected in mental health-related outcomes. Niu et al., using emergency department and hospital encounter data for children, adolescents, and young adults in New York City, found that higher ambient temperature was associated with increased risks of mental health-related emergency department visits or hospitalizations among individuals aged 6–25 years, with elevated risks also observed among adolescents aged 12–17 years (43). Zhang et al., in a case-crossover study conducted in three subtropical cities in China, found that temperature changes were associated with healthcare visits for multiple mental disorders (44). Yoo et al., using a multi-region time-series design in New York, also observed an association between extreme temperatures and emergency department visits related to mental disorders (45). The present study used the CES-D scale to identify a broader level of psychological distress. Taken together, these findings suggest that heat may affect not only physical health but also emotional state and psychological functioning.

Reduced sleep duration may be an important pathway through which extreme heat is associated with depressive symptoms among adolescents. Sleep depends on thermoregulation. Before sleep onset and during sleep, the body needs to reduce core body temperature. Nighttime heat may weaken heat dissipation, increase difficulty falling asleep, and lead to more frequent nighttime awakenings and sleep interruptions. Minor et al., using wearable-device data from 68 countries, found that higher nighttime temperatures shortened sleep duration and increased the risk of insufficient sleep (16). Obradovich et al. also found, based on survey data from the United States, that higher nighttime temperatures were associated with increased sleep insufficiency (46). Sleep compression caused by heat may make adolescents more likely to experience fatigue, irritability, and low mood during the day. In the present study, exploratory pathway analysis showed that the HTD index was negatively associated with log-transformed sleep duration, and log-transformed sleep duration was negatively associated with depressive symptom scores. This finding suggests that heat may be linked to higher depressive symptoms among adolescents by reducing sleep recovery time.

Reduced participation in physical exercise is another important behavioral pathway. Hot weather may change adolescents’ outdoor activity arrangements. Adolescents’ physical exercise often take place in school physical education classes, extracurricular training, playground activities, and community sports spaces. Extreme heat increases the thermal burden and heat-related health risks of outdoor exercise. Schools may adjust physical education classes, parents may reduce children’s outdoor activities, and students themselves may become less willing to exercise. Previous studies have suggested that climate change may alter human physical exercise patterns, and that high-temperature conditions may restrict outdoor activities and exercise participation (18, 19). Physical exercise is also an important protective factor for adolescent mental health. A systematic review and meta-analysis by Recchia et al. found that physical exercise interventions helped reduce depressive symptoms in children and adolescents (32). Li et al., in their study on green space and climate change anxiety, suggested that green physical exercise and environmental comfort are important pathways through which natural environments may influence climate-related mental health, with environmental comfort mediating the association between perceived green space and climate change anxiety (47). In the present study, the HTD index was negatively associated with physical exercise participation, and physical exercise was negatively associated with depressive symptom scores. When heat restricts exercise opportunities, the emotional release, peer interaction, self-efficacy, and physical vitality associated with exercise may be weakened, which may in turn be associated with higher levels of depressive symptoms among adolescents.

Declining self-rated health reflects a more subjective but equally important pathway related to health perception. Fatigue, headache, loss of appetite, insufficient sleep, and impaired attention caused by heat may not always enter the healthcare system, but they can change how adolescents perceive their own health status. Self-rated health is a comprehensive perception of physical condition, energy level, daily functioning, and health risk. Existing studies have shown that self-rated health is closely related to adolescent mental health (34, 35). Kim and Shim, using data from Korean adolescents, also found that subjective health status was associated with stress, suicidal ideation, health behaviors, and physical exercise, suggesting that adolescent self-rated health may serve as an important entry point for school health promotion and early intervention (34). Sawatzky et al. further indicated that adolescents’ self-rated physical health, mental health, and quality of life are closely related (48). The exploratory pathway results of the present study showed that the HTD index was associated with lower self-rated health, and better self-rated health was associated with lower depressive symptom scores. This finding suggests that extreme heat may be linked to increased depressive symptoms by reducing adolescents’ physical comfort and perceived health. Sleep, physical exercise, and self-rated health are not isolated exploratory pathways. Heat disrupts nighttime sleep and reduces daytime energy. Reduced energy and hot weather may further decrease exercise participation. Less exercise and physical discomfort may then further lower perceived health.

Heterogeneity analysis showed that the association between extreme heat exposure and depressive symptoms was more pronounced among rural adolescents. This result is similar to findings from the Chinese adolescent heatwave study, which reported greater vulnerability among rural students (29). Rural adolescents may face more limitations in heat adaptation resources. Some rural households and schools may have relatively limited access to cooling facilities, shaded spaces, indoor sports venues, healthcare services, and mental health support. During hot weather, the sleep environment, learning environment, and exercise environment may all be affected. Urban students are also exposed to heat, but they generally have better access to air conditioning, indoor activity spaces, and health services. Differences in heat exposure across regions are not only differences in temperature, but also differences in resources. This finding suggests that climate adaptation policies should not focus only on average exposure levels; they should also consider differences in adaptive capacity. Rural schools, boarding schools, and adolescents from low-resource families should be priority groups for health protection during hot seasons.

Sex heterogeneity analysis showed that the association between the HTD index and depressive symptoms was more evident among male adolescents. This does not mean that female adolescents are unaffected by heat, nor does it imply that males are inherently more prone to depression. A more plausible explanation may relate to activity patterns and exposure risks. Male adolescents are generally more likely to participate in outdoor sports, high-intensity physical exercise, and peer-based outdoor activities. Heat may therefore have a greater impact on their daily activity rhythms. When outdoor exercise decreases, the emotional release and social interaction originally obtained through exercise may also decline. Hu et al. similarly observed stronger associations between heatwaves and some mental health outcomes among male adolescents, while also noting that sex differences are not entirely consistent across studies (29). Therefore, sex differences should not be simply attributed to biological factors. Social roles, exercise patterns, help-seeking behaviors, emotional expression, and school environments may all be involved. Future studies may further verify these findings by incorporating time-use data, exercise-setting data, and mental health service-seeking behaviors.

The exploratory pathway results also suggest that adolescents with poorer self-rated health may require particular attention. Adolescents with weaker perceived health may be more likely to experience fatigue, sleep disturbance, reduced activity capacity, and physical discomfort when facing high temperatures. Physical discomfort may reduce learning status and life satisfaction and may also increase negative emotions. Existing evidence suggests that the mental health effects of temperature changes may be jointly moderated by physiological sensitivity, family resources, and adaptive capacity (7, 26). The present findings further indicate that, within the adolescent population, perceived health itself may serve as an important signal of vulnerability. During hot seasons, schools and families should not focus only on students with diagnosed diseases, but should also pay attention to students with poorer self-rated health, frequent fatigue, insufficient sleep, and low levels of physical exercise.

This study has several strengths. First, the study focused on Chinese adolescents, helping to address the lack of age-specific evidence in climate change and mental health research. Second, this study used multiple waves of CFPS data and matched them with the Chinese Climate Physical Risk Index, making it possible to examine the association between extreme heat exposure and adolescent depressive symptoms within a national sample framework. Third, this study not only examined the main association but also explored possible behavioral and health-related pathways involving three modifiable factors: sleep duration, physical exercise, and self-rated health. It also identified rural and male adolescents as key groups and further suggested that adolescents with poorer self-rated health, insufficient sleep, and lower levels of physical exercise deserve greater attention. These findings provide evidence for stratified interventions by schools, families, and public health agencies (49).

This study also has several limitations. First, because of limited access to detailed geographic information in the public CFPS data, this study matched climate exposure using the province-year-level HTD index. This approach captures regional relative extreme heat risk but cannot accurately reflect individual-level heat exposure, local microclimates, school environments, housing conditions, air-conditioning use, outdoor activity time, or personal protective behaviors. Given the substantial geographic heterogeneity within Chinese provinces, exposure misclassification may exist. Therefore, the findings should be interpreted as associations between regional extreme heat risk and adolescent depressive symptoms, rather than as precise estimates of individual heat exposure effects. Future studies should use finer-scale meteorological data, such as city-, county-, or grid-level temperature data, combined with detailed residential, school, and behavioral information to improve exposure assessment. Second, the CES-D measures depressive symptoms rather than clinical diagnosis. It is suitable for population-based research but cannot replace psychiatric diagnosis. Third, although this study controlled for province fixed effects, year fixed effects, and a series of individual and family variables, and conducted several robustness checks, the observational design still cannot fully rule out unobserved confounding. Fourth, sleep duration, physical exercise, self-rated health, and depressive symptoms may have bidirectional relationships. Therefore, the exploratory pathway analysis in this study was mainly used to identify potential pathways and should not be interpreted as evidence of strict causal mediation. Future studies could combine finer-scale meteorological data, wearable devices, diary surveys, school environmental data, and quasi-experimental designs to further reveal the dynamic process through which extreme heat affects adolescent mental health.

These findings also have several practical implications. First, schools should adjust physical education schedules during periods of extreme heat, such as moving outdoor classes to cooler morning or late-afternoon hours and providing indoor exercise alternatives when necessary. Second, improving sleep environments during hot seasons may help reduce heat-related psychological risks, especially for adolescents in rural or low-resource settings. Third, rural schools and communities should strengthen cooling facilities, shaded activity spaces, and access to safe indoor physical exercise spaces. Finally, adolescents with insufficient sleep, low participation in physical exercise, or poorer self-rated health may require targeted psychological screening and early support during extreme heat events.

## 5 Conclusion

This study found that extreme heat exposure was associated with higher levels of depressive symptoms among Chinese adolescents. Shorter sleep duration, reduced participation in physical exercise, and lower self-rated health may represent important behavioral and health-related pathways underlying this association. Rural and male adolescents may be more vulnerable to the mental health risks associated with extreme heat. Adolescents with poorer self-rated health, insufficient sleep, and lower levels of physical exercise should also receive particular attention.

These findings suggest that, in the context of global warming and increasingly frequent extreme heat events, adolescent mental health protection should be incorporated into school health promotion, family education, and public health climate adaptation policies. Extreme heat should not be viewed only as a physical health risk, but also as a complex environmental stressor that may alter adolescents’ sleep, physical exercise, health perceptions, and emotional states.

## Data Availability

The individual-level data were obtained from the China Family Panel Studies (CFPS), conducted by the Institute of Social Science Survey, Peking University. Data repository: CFPS Data Center/Peking University Open Research Data Platform. Direct links: https://cfpsdata.pku.edu.cn/#/home and https://opendata.pku.edu.cn/dataverse/CFPS. The climate exposure data were obtained from the Chinese Climate Physical Risk Index (CCPRI). Data repository: Climate Risk Database/CPRI dataset. Direct links: https://www.cnefn.com/data/download/climate-risk-database/ and https://figshare.com/articles/dataset/Climate_Physical_Risk_Index_CPRI_/25562229.
